# AI-Driven Polymeric Coatings: Strategies for Material Selection and Performance Evaluation in Structural Applications

**DOI:** 10.3390/polym18010005

**Published:** 2025-12-19

**Authors:** Min Ook Kim

**Affiliations:** Department of Civil Engineering, Seoul National University of Science and Technology, 232 Gongneung-ro, Nowon-gu, Seoul 01811, Republic of Korea; minookkim@seoultech.ac.kr

**Keywords:** artificial intelligence, polymeric coatings, material selection, performance evaluation

## Abstract

Polymeric coatings play a pivotal role in enhancing the durability, functionality, and sustainability of structural materials exposed to harsh environmental conditions. Recent advances in artificial intelligence (AI) have transformed the development, optimization, and evaluation of these coatings by enabling data-driven material discovery, predictive performance modeling, and autonomous inspection. This review aims to provide a comprehensive overview on AI-driven polymeric coating strategies for structural applications, emphasizing the integration of machine learning, computer vision, and multi-physics simulations into traditional materials engineering frameworks. The discussion encompasses AI-assisted material selection methods for polymers, fillers, and surface modifiers; predictive models for corrosion, fatigue, and degradation; and intelligent evaluation systems using digital imaging, sensor fusion, and data analytics. Case studies highlight emerging trends such as self-healing, smart, and sustainable coatings that leverage AI to balance mechanical performance, environmental resistance, and carbon footprint. The review concludes with identifying current challenges—including data scarcity, model interpretability, and cross-domain integration—and proposes future research directions toward explainable, autonomous, and circular coating design pipelines.

## 1. Introduction

The durability and reliability of modern infrastructure rely heavily on protective coatings that serve as the first line of defense against mechanical wear, corrosion, chemical attack, and environmental degradation. Structural materials such as steel, concrete, and fiber-reinforced composites (FRCs) are routinely exposed to aggressive conditions—salt spray, moisture, ultraviolet (UV) radiation, freeze–thaw cycles, and mechanical impact—that progressively deteriorate their performance and shorten service life [1]. As maintenance and replacement costs rise globally, the need for high-performance, long-lasting, and environmentally benign coating systems has become increasingly urgent. Among the wide variety of surface protection technologies, polymeric coatings have emerged as indispensable due to their superior processability, chemical versatility, and capability for tailored functionality through molecular design and hybridization [2].

Polymeric coatings are typically composed of organic binders, pigments, fillers, and additives engineered to impart barrier, adhesion, and self-healing properties. Epoxy, polyurethane, acrylic, fluoropolymer, and silicone systems dominate current structural applications, while hybrid organic–inorganic architectures are gaining traction for enhanced thermal stability and chemical resistance. The versatility of polymeric coatings allows their use across diverse structural contexts—from steel bridges, pipelines, and offshore platforms to concrete facades and composite reinforcements [3]. Beyond conventional anti-corrosion or anti-fouling roles, the next generation of coatings is increasingly multifunctional—capable of self-sensing, self-cleaning, and responding adaptively to mechanical or environmental stimuli [4]. However, designing such coatings might involve highly complex relationships between material constituents, processing conditions, and service environments, often spanning multiple physical and temporal scales.

**Figure 1:**
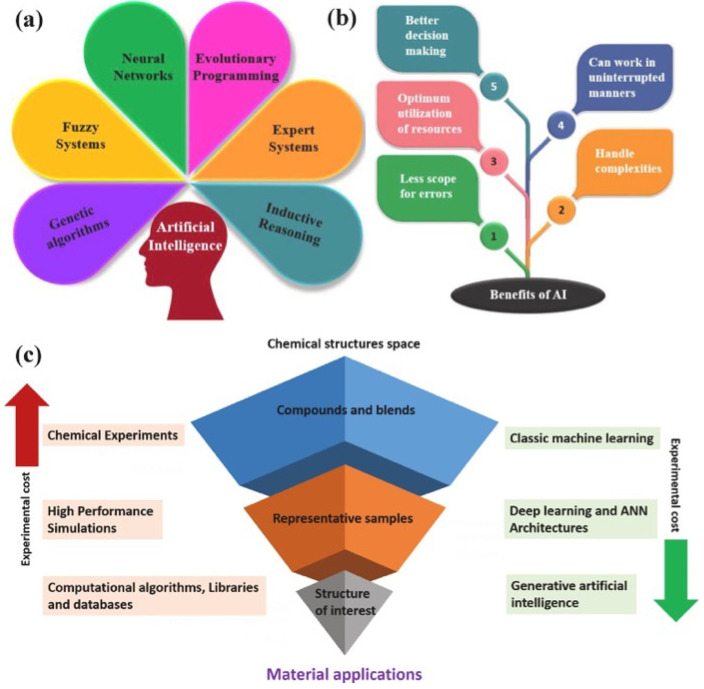
(**a**) Components of AI; (**b**) Advantages of AI technique; (**c**) Computerized and AI-equipped approaches in materials discovery, reprinted from Ref. [10].

Traditionally, coating development has mainly relied on empirical or semi-empirical methodologies. Formulations are iteratively adjusted through experimental trial-and-error, guided by limited domain knowledge or heuristic intuition [5,6]. However, this kind of approach could be time-consuming, costly, and insufficient to meet the accelerating demands of modern structural engineering. This is because the number of possible formulations and processing parameters grows with each added component or environmental variable [7]. Furthermore, many key performance indicators—such as corrosion initiation, adhesion degradation, or fatigue cracking—are governed by nonlinear interactions and time-dependent processes that are difficult to capture with conventional deterministic models [8,9]. In recent years, however, the rapid evolution of AI and machine learning (ML) has opened unprecedented opportunities to revolutionize materials design and evaluation, as illustrated in Figure 1 [5,6,10].

AI-driven approaches can discover hidden correlations within large, multi-modal datasets, enabling the prediction and optimization of complex material behaviors that are otherwise intractable [11,12]. In the context of polymeric coatings, importantly, AI can be leveraged across every stage of the materials development cycle: from molecular design and formulation optimization to performance monitoring and lifecycle assessment [13]. For example, supervised learning algorithms such as random forests and gradient-boosting models have been employed to predict coating adhesion strength, corrosion rate, and hardness from compositional descriptors [14,15]. Deep neural networks can model the nonlinear degradation kinetics of coatings under variable humidity or temperature [16]. Reinforcement learning and Bayesian optimization frameworks are emerging to autonomously guide experimental design, suggesting optimal combinations of polymer matrices, fillers, and crosslinkers based on performance targets and cost constraints [17,18,19].

Recent literature provides clear and experimentally validated examples of AI-driven polymeric coating design [15,20,21,22]. Karuth et al. presented an integrated machine-learning, computational, and experimental framework to evaluate compatibility in oil-modified silicone elastomer coatings, where ML models identified key molecular and formulation descriptors governing coating compatibility, and predictions were verified through experimental characterization [20]. Yan et al. employed multiple machine-learning algorithms to predict the tribological performance of epoxy composite coatings based on formulation and testing parameters, demonstrating close agreement between the predicted and measured friction and wear data [15]. Chen et al. developed a two-stage machine-learning approach linking environmental exposure conditions to physical property evolution and ultimately to corrosion failure, using experimentally obtained degradation data to train and validate the models, thus enabling realistic service-life prediction of coatings [21]. Liu et al. reported a machine-learning-assisted discovery of high-efficiency self-healing epoxy coatings, where AI-guided formulation screening led to optimized coating systems that were subsequently confirmed through electrochemical corrosion tests and self-healing performance evaluations [22]. Collectively, these studies demonstrate that AI-based coating design is no longer conceptual, but is increasingly supported by rigorous experimental validation and real-world performance assessment.

Despite these promising advances, several fundamental challenges still remain. A major limitation is the scarcity and heterogeneity of high-quality datasets, as coating research often involves proprietary industrial data or small-scale academic studies with inconsistent testing protocols. In addition, model interpretability remains a concern; black-box AI predictions are often difficult to rationalize in terms of underlying chemical or physical mechanisms, impeding scientific understanding and regulatory acceptance. The lack of standardized data schemas, interoperable ontologies, and cross-disciplinary collaboration further constrains progress. Bridging these gaps requires not only computational sophistication but also the integration of materials science knowledge, experimental validation, and engineering practice into coherent AI-driven frameworks. In this regard, this study aims to provide a comprehensive and systematic perspective on AI-driven polymeric coating strategies and methods for structural applications, with particular emphasis on material selection and performance evaluation. First, this review surveys the state-of-the-art polymeric coating systems and their functional requirements across structural engineering sectors, highlighting the limitations of conventional formulation and testing methods. Second, it elucidates the application of AI and machine learning techniques to coating material selection—covering data-driven polymer design, additive screening, and microstructure optimization. Third, it examines AI-enabled evaluation and monitoring techniques, including vision-based defect recognition, sensor-data analytics, and predictive modeling of degradation behavior. Finally, the review identifies major technical and conceptual challenges, such as data standardization, model explainability, and scalability, and proposes future research directions toward autonomous coating design pipelines integrating experimentation, simulation, and field feedback.

## 2. Current Challenges in Conventional Coating Development

Polymeric coatings are essential for extending the service life and reliability of structural materials exposed to aggressive mechanical, chemical, and environmental conditions, primarily by acting as barrier layers against moisture, chloride ions, UV radiation, and industrial pollutants [23,24]. Their protective performance arises from complex interactions among multiple formulation variables—including polymer binders, pigments, fillers, and functional additives—that collectively govern adhesion, film formation, mechanical integrity, and durability [25,26,27]. Over time, coating systems such as epoxy, polyurethane, acrylic, fluoropolymer, silicone, and hybrid organic–inorganic formulations have become widely adopted in structural and industrial applications due to their tailored property profiles [28,29,30,31,32,33] (see Table 1).

Despite this maturity, the development of high-performance polymeric coatings has traditionally relied on empirical, trial-and-error-based formulation strategies. In this conventional paradigm, coating performance is optimized through repeated experimental adjustments of composition, followed by standardized qualification testing. While industry standards established by organizations such as ASTM, ISO, and NACE provide essential benchmarks for corrosion resistance, adhesion, mechanical durability, and weathering performance, they primarily evaluate macroscopic responses such as blistering, rust formation, gloss loss, or adhesion failure [34,35,36,37,38,39] (See Table 2). These tests offer limited insight into the underlying molecular-scale and interfacial degradation mechanisms that ultimately control long-term performance.

A fundamental challenge of this traditional approach is the strong nonlinearity and coupling between formulation variables and performance outcomes. Small changes in binder chemistry, filler dispersion, curing conditions, or additive concentration can lead to disproportionate and sometimes counterintuitive effects on durability, adhesion, or environmental resistance. As a result, systematic exploration of the vast compositional design space is impractical using purely experimental methods. Optimization therefore proceeds incrementally, requiring numerous formulation–testing cycles that are both time-consuming and resource-intensive [40,41,42].

Moreover, conventional evaluation strategies are largely retrospective rather than predictive. Accelerated laboratory tests and long-term field exposure studies—often spanning months or years—provide valuable validation of coating performance under specific conditions [43,44,45], but they offer limited capability to forecast degradation trajectories under variable or coupled service environments. Analytical techniques such as FTIR, SEM, DSC, TGA, and EIS can characterize chemical and microstructural changes [46,47], yet the resulting data are typically interpreted qualitatively or in isolation, rather than integrated into unified, predictive design frameworks.

These limitations are further compounded by fragmented data generation and the lack of systematic digitalization, which hinder knowledge accumulation and reuse across projects. In addition, sustainability considerations—such as VOC reduction, material efficiency, and lifecycle environmental impact—are difficult to optimize concurrently with performance when relying solely on empirical development strategies.

In summary, while conventional coating development and standardized testing have established a robust foundation for structural protection, they are inherently constrained by nonlinear formulation–performance relationships, high experimental costs, and limited predictive capability. These challenges underscore the need for data-driven and AI-enabled methodologies capable of efficiently navigating complex design spaces, integrating heterogeneous datasets, and enabling predictive optimization of next-generation polymeric coating systems.

## 3. AI-Driven Polymeric Coatings: Material Selection

The integration of AI and ML into polymeric coating research marks a decisive shift from empirical trial-and-error formulation toward predictive, data-centric design [48,49,50]. Conventional coating development—based on incremental modification of binders, pigments, and additives—has evolved into an intelligent, algorithm-assisted process capable of linking chemical structure, microstructure, and macroscopic performance within a unified digital framework [51,52]. This means AI not only accelerates discovery but also enhances scientific understanding by capturing nonlinear relationships and multiscale dependencies that are often inaccessible through conventional modeling or intuition.

Coating performance in structural applications is governed not only by material formulation but also by the selection of an appropriate application technique [53,54,55,56,57,58,59]. Industrial methods such as airless spraying, roller coating, and dipping/immersion coating differ substantially in atomization behavior, achievable film thickness, rheological demands, and process constraints, as shown in Table 3.

Airless spraying is widely employed for steel and concrete infrastructure because it provides high transfer efficiency and uniform films over large areas. Its performance is strongly influenced by viscosity, solids content, and thixotropy, which control atomization quality and droplet size [53]. AI-driven prediction of these parameters enables the identification of optimal formulation windows that prevent defects such as orange peel, overspray, and sagging. AI-assisted rheology optimization further supports rapid tuning of binders and additives to maintain stable spraying across varying nozzle pressures and environmental conditions [54]. Roller coating is preferred when consistent film thickness and minimal material loss are required. This method tolerates higher viscosity and solids content, making it suitable for thick, protective layers on industrial floors and concrete surfaces. AI-based predictions of viscoelasticity, leveling behavior, and open time help mitigate ridging, unevenness, and other surface defects while enabling more precise control of formulation–process interactions [55,56]. Dipping/immersion coating is commonly used for complex geometries and fully exposed components, including reinforcement bars and marine hardware. Successful application requires low viscosity, strong wetting, and adequate drain-time before gelation. AI-enabled prediction of surface energy, wetting indices, and cure kinetics improves control over drainage behavior and reduces defects such as runs, curtaining, and thickness variations. Recent ML studies demonstrate strong capability in predicting viscosity and cure behavior critical to immersion-coating performance [57,58].

With recent advances in AI, key formulation parameters—viscosity, solids content, thixotropy, wetting behavior, open time, and cure time—can now be predicted with high fidelity from molecular descriptors and experimental datasets. These insights allow AI systems to recommend not only optimized formulations but also the most suitable application technique and corresponding operational settings (e.g., nozzle pressure, roller hardness, immersion duration). Consequently, AI provides an integrated framework linking material design and process engineering, enhancing film quality, reducing waste, improving reproducibility, and enabling proactive defect prevention in industrial coating operations [59].

**Figure 2:**
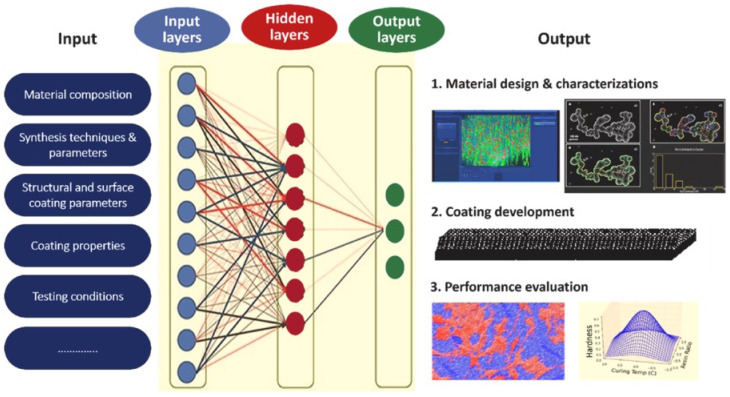
AI technique for multifunctional coating development, reprinted from Ref. [10].

At the core of AI-driven polymeric coating design lies the translation of materials chemistry into machine-readable descriptors [10,60]. Specifically, polymer composition, molecular topology, and interfacial characteristics can be encoded as numerical or graph-based representations, which serve as the foundation for predictive modeling [61]. These descriptors include monomer type, cross-link density, degree of polymerization, polarity indices, glass-transition temperature, and thermodynamic solubility parameters, among others [62]. By learning from large datasets of measured or simulated properties, ML algorithms such as random forests, support-vector regression, Gaussian processes, and deep neural networks can predict performance indicators—including adhesion energy, corrosion resistance, diffusion coefficients, and weathering durability—directly from the formulation parameters [63,64,65,66]. This approach enables virtual screening of candidate materials long before laboratory synthesis, thereby reducing experimental iterations and resource consumption, as shown in Figure 2. Table 4 shows the summary of AI/ML algorithms in polymeric coatings.

A particularly powerful development has recently been the application of graph-based and transformer neural networks for polymer informatics [67,68]. These architectures represent polymers as molecular graphs or sequence embeddings, allowing the model to interpret chemical connectivity, functional groups, and intermolecular interactions directly. To be specific, both graph convolutional networks (GCNs) and message-passing neural networks (MPNNs) have demonstrated remarkable accuracy in predicting polymer mechanical properties, barrier efficiency, and chemical stability [67,68]. When applied to coating design, such models facilitate the rapid identification of resin systems or crosslinkers that optimize both mechanical flexibility and environmental resistance, an optimization that has historically required extensive manual experimentation.

In parallel, generative AI models have opened new avenues for inverse design and autonomous material discovery [69,70,71]. Specifically, variational autoencoders (VAEs), generative adversarial networks (GANs), and diffusion models can generate novel polymer backbones, copolymer ratios, and additive combinations that meet predefined target properties such as low water permeability or enhanced UV absorption. By coupling these generative models with high-throughput simulation tools—such as molecular dynamics (MD) or finite-element (FE) models—researchers can establish closed-loop frameworks for property-driven optimization [72,73]. In this closed loop, AI algorithms propose new molecular structures, computational models predict their expected performance, and reinforcement-learning agents refine the search strategy based on performance feedback. This iterative, autonomous cycle dramatically accelerates innovation in coating materials, achieving in weeks what once required years of empirical experimentation.

Material selection in AI-driven coating design extends beyond polymer matrices to encompass fillers, nanoparticles, pigments, and hybrid modifiers, all of which profoundly influence barrier and mechanical behavior. Multi-objective optimization algorithms, such as Bayesian optimization and genetic algorithms, are used to balance competing design goals—for example, maximizing corrosion resistance while minimizing cost and environmental impact [74,75]. Through data fusion of experimental databases, quantum-chemical simulations, and microstructural characterization, AI can identify synergistic material combinations such as graphene-oxide-reinforced epoxies, silane-functionalized polyurethanes, or nano-TiO_2_-modified acrylics that deliver superior protection through tailored microstructures [49,76]. Furthermore, ML-based clustering and dimensionality-reduction techniques assist in classifying material families and mapping performance trends across the vast compositional space of polymeric coatings [49,77].

An emerging domain of particular relevance to sustainable infrastructure is the integration of AI-assisted lifecycle assessment (LCA) and environmental performance metrics into material selection [78]. By correlating structural durability predictions with embodied energy, CO_2_ footprint, and recyclability indices, intelligent systems can recommend coating materials that achieve both technical and sustainability targets. Reinforcement-learning algorithms, for example, can iteratively adjust formulation parameters to minimize VOC emissions or optimize curing temperature while maintaining desired barrier performance [79]. This holistic approach aligns AI-enabled design not only with engineering performance but also with global decarbonization and circular-economy goals.

**Figure 3:**
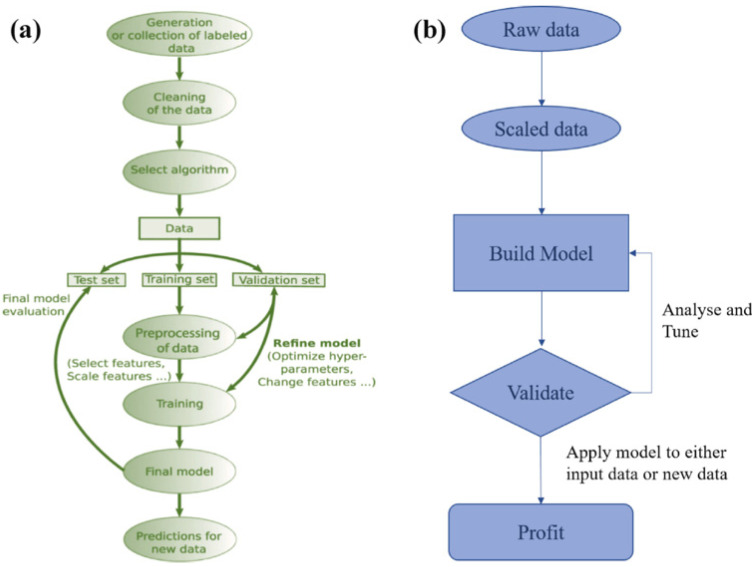
(**a**) Supervised learning workflow; (**b**) Unsupervised learning workflow. Reprinted from Refs. [10,80].

The practical implementation of AI-driven coating design is increasingly supported by materials databases and digital infrastructures, as shown in Figure 3 [10,60,80]. Some existing platforms such as Polymer Genome, ChemML, Citrination, and the Materials Project provide structured data on polymer properties, molecular structures, and processing conditions. 

These resources, combined with laboratory-specific datasets curated from experimental results, enable transfer learning—where a pretrained model developed on one class of polymers can be fine-tuned to predict behavior in another. The establishment of interoperable ontologies and standardized data schemas ensures that diverse experimental and computational datasets can be integrated seamlessly, supporting collaborative research and reproducible AI modeling.

A central advantage of AI-driven material selection is its ability to account for multiscale coupling effects inherent to coating systems [81]. Coating performance arises from interactions across molecular, microstructural, and macroscopic levels: chain entanglement and crosslinking define local stiffness, filler dispersion governs micro-barrier efficiency, and interfacial adhesion controls macroscopic durability. Traditional models often isolate these phenomena, whereas AI can integrate heterogeneous data—from atomistic simulations, spectroscopy, microscopy, and full-scale field tests—into unified predictive frameworks. By learning the correlations across scales, AI models can forecast long-term degradation, crack initiation, and interfacial delamination under variable service conditions, thereby informing more reliable material selection and system design.

Nevertheless, the successful deployment of AI-driven polymeric coating design requires careful attention to data curation, model interpretability, and physical validation [82]. Experimental reproducibility, data sparsity, and variations in testing protocols can introduce bias into training datasets. Moreover, purely data-driven predictions may overlook physically meaningful mechanisms. To address these challenges, researchers increasingly employ physics-informed machine learning (PIML), which embeds governing equations—such as Fickian diffusion, reaction kinetics, or viscoelastic constitutive laws—within neural network architectures. This hybrid approach ensures that predictions remain consistent with known material behavior, improving both generalization and scientific credibility [83]. Similarly, explainable AI (XAI) techniques, such as SHAP values and attention mapping, provide transparency into feature importance, allowing researchers to interpret which compositional or structural attributes most strongly influence coating performance [84].

In sum, AI-driven polymeric coating design and material selection represent a paradigm shift from empirical exploration to rational, data-driven engineering. Through predictive modeling, generative synthesis, and multi-objective optimization, AI enables the rapid discovery of coating systems that simultaneously meet the mechanical, environmental, and sustainability requirements. By uniting chemistry, mechanics, and informatics within a digital design loop, this approach transforms coatings from passive protective layers into intelligently engineered materials—tailored, adaptive, and optimized for the next generation of resilient and sustainable infrastructure.

## 4. AI-Driven Performance Evaluation and Predictive Degradation Modeling

As discussed in previous sections, the performance evaluation of polymeric coatings has traditionally relied on standardized laboratory tests and long-term field exposure programs. While these approaches provide essential baseline information, they often struggle to capture the complex, time-dependent coupling of mechanical, chemical, and environmental degradation mechanisms that govern real-world coating failure [46]. In this context, AI and ML offer a paradigm shift toward data-centric, predictive modeling frameworks capable of forecasting coating degradation and service life with enhanced accuracy [85,86].

At the core of AI-driven performance evaluation is data acquisition and integration focused on degradation-relevant variables [87,88]. Modern coating systems increasingly generate multivariate datasets from electrochemical impedance spectroscopy (EIS), acoustic emission (AE), fiber-optic strain sensing, and environmental monitoring. These datasets provide continuous time-series records of impedance evolution, moisture ingress, stress accumulation, and chemical change, forming a digital representation of coating condition throughout exposure. ML models trained on such data can identify latent degradation signatures that precede visible damage, enabling the early prediction of blistering, delamination, or corrosion initiation.

AI-based time-dependent predictive modeling plays a central role in translating monitoring data into degradation curves and lifetime estimates. Recurrent neural networks (RNNs), particularly long short-term memory (LSTM) architectures, have demonstrated strong capability in capturing nonlinear temporal dependencies in coating aging, cyclic loading, and environmental exposure histories. Similarly, convolutional neural networks (CNNs) and hybrid CNN–RNN models have been applied to sequential signal data and image-derived degradation indicators to quantify progression rates and predict failure thresholds under variable conditions.

In parallel, AI-assisted image and signal analytics enhance the objectivity and consistency of laboratory and field performance evaluation. Computer-vision models trained on annotated surface images can automatically detect and quantify cracks, pinholes, chalking, rust formation, and other degradation features with reduced operator bias [89,90]. For example, deep learning models trained on annotated inspection datasets have demonstrated high detection performance in practical defect identification tasks. A Faster R-CNN model achieved a mean average precision of 94.9% in rail surface defect detection [91] Likewise, CNN-based corrosion classification approaches have exceeded 95% accuracy on engineered corrosion datasets [92]. In contrast, traditional computer vision and manual inspection methods generally yield lower accuracies (e.g., 69 to 78% in corrosion classification benchmarks), underscoring the performance advantages of deep learning-based inspection [93]. Beyond classification, these models enable quantitative estimation of defect size, spatial density, and morphological evolution, thereby significantly reducing subjectivity and improving reproducibility in quality control processes.

**Figure 4:**
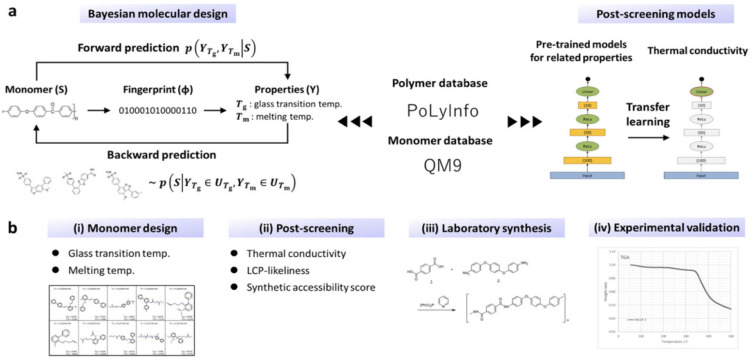
ML-assisted de novo design and experimental validation of new polymers. (**a**) The objective of forward prediction is to derive a model that describes polymeric properties; (**b**) Analytic workflow consisting of four internal steps towards materials discovery, reprinted from Ref. [96].

A significant advancement in this domain is the development of predictive degradation models that integrate experimental data with mechanistic understanding [94]. ML algorithms trained on both experimental and simulated datasets can approximate nonlinear degradation pathways and continuously update predictions as new data become available [95]. Hybrid physics–ML models, for example, combine neural networks with diffusion–reaction or viscoelastic damage formulations, enabling coating lifetime prediction under coupled temperature, humidity, and mechanical loading. Bayesian inference frameworks further enhance reliability by quantifying uncertainty and adaptively updating model parameters as monitoring data are accumulated (see Figure 4) [96].

AI-driven predictive modeling also enables accelerated qualification and virtual aging assessment [97]. By learning from historical exposure datasets, ML models can extrapolate long-term degradation trends from short-duration accelerated tests, substantially reducing experimental time and cost. Transfer learning techniques allow predictive models developed for one coating system or exposure condition to be adapted to new formulations with minimal retraining, supporting rapid screening and certification of eco-friendly or low-VOC coatings.

Despite these advances, challenges remain. High-fidelity prediction depends on the availability of standardized, high-quality datasets spanning diverse materials, environments, and testing protocols. Sensor noise, data gaps, and dataset imbalance can impair model robustness, while purely data-driven approaches risk overfitting or obscuring causal mechanisms. To address these limitations, physics-informed machine learning (PIML) and explainable AI (XAI) approaches are increasingly adopted [98]. By embedding governing principles—such as Fickian diffusion, electrochemical corrosion kinetics, and viscoelastic creep—into learning architectures, PIML improves physical consistency and interpretability. XAI techniques further clarify which variables most strongly influence the predicted degradation outcomes, enhancing trust in AI-assisted prognostics.

Overall, AI-driven performance evaluation and predictive degradation modeling represent a decisive transition from empirical, retrospective assessment toward intelligent prognostics at the material level. By leveraging time-series learning, uncertainty-aware prediction, and physics integration, these approaches enable early failure detection, reliable lifetime estimation, and data-driven coating optimization—forming the foundation for higher-level system integration discussed in the following section.

## 5. Integration of AI-Driven Coating Design with Digital Twin and Structural Health Monitoring Systems

Building upon material-level predictive models, the integration of AI-driven coating design with DT and structural health monitoring (SHM) systems extends performance assessment from isolated material behavior to system-wide asset management. In this framework, coatings are no longer treated as standalone protective layers but as active components within a cyber–physical infrastructure ecosystem [99,100,101].

SHM systems employ distributed sensor networks—including strain gauges, acoustic emission sensors, fiber Bragg gratings, corrosion potential probes, and environmental sensors—to collect multiscale data from critical structural regions [102]. AI algorithms process these heterogeneous datasets to detect anomalies, estimate remaining useful life (RUL), and evaluate the structural implications of coating degradation. For coated steel bridges, offshore platforms, and marine concrete facilities, this integration enables precise localization of coating damage, quantification of degradation rates, and real-time assessment of risk propagation from material failure to structural performance [103].

Digital twins provide the system-level integration layer that unifies material degradation models, SHM data, and structural simulations into a continuously evolving virtual replica of the physical asset [104]. Material-scale AI models predict the evolution of coating properties—such as permeability, adhesion strength, and microcrack density—under environmental exposure, while SHM data on strain, vibration, and loading conditions feed back into the twin to simulate stress-assisted degradation mechanisms. This bidirectional coupling establishes a closed-loop cyber–physical system in which monitoring data update models, models inform decisions, and interventions mitigate failure [104,105].

Practically, AI-enabled DT architectures typically consist of four layers: (1) a physical layer of sensors and monitoring hardware, (2) a data layer for real-time acquisition and preprocessing, (3) an AI-analytics layer for feature extraction, anomaly detection, and prediction, and (4) a decision layer interfacing with maintenance and asset-management systems. Cloud and edge computing infrastructures ensure scalability and responsiveness, while IoT frameworks enable seamless connectivity among coating sensors, SHM devices, and DT platforms—even in remote or marine environments [106,107,108].

The predictive power of these integrated systems is further enhanced through hybrid modeling, combining AI-based analytics with mechanistic models of diffusion, corrosion, and fatigue [109]. Reinforcement-learning algorithms can optimize inspection intervals and maintenance strategies by learning from historical degradation and repair records, resulting in self-adaptive maintenance ecosystems that balance reliability, cost efficiency, and sustainability.

A key advantage of AI–DT–SHM integration lies in cross-scale knowledge transfer [94]. Information generated during coating design—such as predicted molecular stability, filler dispersion, and interfacial adhesion—can be embedded into DTs as prior knowledge, improving degradation simulations. Conversely, field data collected through SHM continuously refine AI models for next-generation coating design, creating a closed-loop innovation cycle linking laboratory, simulation, and real-world performance.

Early implementations of this vision are emerging in infrastructure and energy systems [110]. For example, DTs of offshore wind turbine towers incorporating AI-based coating degradation models have demonstrated improved prediction of corrosion under biofouling and tidal exposure [111]. Similarly, bridge management systems integrating AI-enhanced SHM with coating DTs have reported maintenance cost reductions of up to 22% by enabling condition-based rather than schedule-based interventions [112].

Despite these successes, challenges remain, including data interoperability, sensor standardization, computational demands, and the need for transparent, trustworthy AI predictions in safety-critical applications. Advances in XAI, uncertainty quantification, and physics-informed modeling are essential to overcome these barriers and ensure broad adoption.

In summary, the integration of AI-driven coating design with DT and SHM systems represents a decisive step toward intelligent, sustainable infrastructure management. By uniting material-level predictive modeling with system-level cyber–physical integration, these frameworks enable proactive maintenance, lifecycle optimization, and resilience enhancement—transforming polymeric coatings from passive barriers into intelligent, adaptive interfaces within smart infrastructure systems.

## 6. Limitations and Future Works

The advancement of AI, ML, and digital technologies has reshaped the design, evaluation, and maintenance of polymeric coating systems. However, despite remarkable progress, the translation of AI-driven methods from conceptual demonstrations to widespread industrial practice remains an ongoing challenge. The future of AI-enabled coating science will depend on overcoming several key barriers—data quality, interpretability, integration, and sustainability—while embracing emerging opportunities such as autonomous experimentation, self-adaptive materials, and carbon-neutral innovation.

A primary challenge in the adoption of AI-driven coating systems lies in data availability and standardization. Reliable model training requires large, diverse, and high-quality datasets encompassing chemical compositions, environmental conditions, and long-term performance metrics. However, the coating industry is characterized by fragmented proprietary databases, inconsistent experimental protocols, and limited access to long-term field data [113]. These constraints hinder the generalization and transferability of AI models. Addressing this issue will require global efforts to establish open, standardized data infrastructures for coating materials—complete with metadata, uncertainty quantification, and ontology frameworks linking molecular, microstructural, and macroscopic descriptors [114].

A second critical issue involves model interpretability and scientific transparency. While deep neural networks and generative models can accurately predict performance or generate new formulations, they often function as opaque black boxes, offering limited physical insight. This poses a fundamental barrier to scientific validation and regulatory acceptance, particularly for safety-critical applications in infrastructure and marine environments. The adoption of XAI and physics-informed machine learning (PIML) represents a promising solution. By embedding physical constraints, thermodynamic laws, or diffusion-reaction equations directly within learning architectures, these approaches ensure that AI predictions remain consistent with known material behavior. Moreover, interpretability tools such as SHAP, Grad-CAM, and attention mapping can identify which molecular features, compositional parameters, or environmental variables exert the greatest influence on coating performance. The fusion of data-driven inference with physical interpretability will be essential to transform AI from a predictive tool into a scientific discovery engine.

A third challenge concerns cross-disciplinary integration across the coating value chain—from molecular design to structural deployment. Bridging the scales that connect molecular chemistry, microstructural evolution, and macroscopic mechanical behavior remains a formidable task. AI models trained exclusively at one scale often fail to capture the emergent phenomena governing real-world degradation, such as stress-assisted delamination or moisture-ion coupling [115]. Progress will require hybrid frameworks that unify multiscale simulations (e.g., molecular dynamics, coarse-grained modeling, FE analysis) with experimental data from spectroscopy, microscopy, and full-scale field testing. Such strategies will enable the development of digital twins that not only simulate coating behavior but also evolve dynamically as new monitoring data become available. In parallel, the integration of SHM data will further enhance the ability of AI models to link material performance directly with structural reliability and maintenance planning.

Beyond the technical challenges summarized above, the ethical and environmental dimensions of AI-enabled materials innovation warrant careful consideration. The growing computational demands of deep-learning models contribute to non-negligible energy consumption and carbon emissions, necessitating greener computation strategies and sustainable digital infrastructure. Simultaneously, AI offers unprecedented opportunities to reduce environmental impact through the optimization of coating formulations for lower VOC emissions, recyclability, and extended service life. AI-assisted LCA tools can evaluate trade-offs among environmental burden, performance, and cost, guiding the design of truly sustainable coating systems. The integration of sustainability metrics into AI frameworks will be crucial to align scientific innovation with global decarbonization targets and circular-economy principles.

From an opportunity perspective, autonomous and self-driving laboratories are emerging as transformative platforms for next-generation coating research [116]. By coupling robotic synthesis, automated characterization, and reinforcement-learning optimization, these systems can conduct closed-loop experimentation where AI autonomously selects, prepares, and evaluates new coating formulations. This paradigm promises orders-of-magnitude acceleration in materials discovery while ensuring reproducibility and data integrity. The combination of autonomous laboratories with cloud-connected digital twins could ultimately enable continuous learning ecosystems where real-world performance data directly inform future material design cycles.

The future landscape of AI-enabled polymeric coatings also points toward intelligent and adaptive materials—coatings that can sense, respond, and heal in real-time. Embedded sensor networks, conductive fillers, and smart polymers capable of reversible crosslinking or stimuli-responsive behavior (e.g., self-healing through thermal or chemical triggers) can be integrated with AI-based control algorithms. Such cognitive coatings will no longer be passive barriers but active interfaces that interact with their environment, diagnose their condition, and initiate self-repair. The convergence of materials informatics, additive manufacturing, and smart polymer chemistry will thus redefine protective coatings as cyber-physical systems embedded within the broader IoT and smart-infrastructure ecosystems.

The integration of AI into polymeric coating science represents a paradigm shift from empirical design and static testing to intelligent, self-optimizing, and sustainable materials engineering. The convergence of AI, DT, and SHM will enable real-time prediction, adaptation, and maintenance across the entire lifecycle of structural coatings. As these technologies mature, the next generation of polymeric coatings will evolve from inert protective layers to autonomous, learning, and responsive systems—an embodiment of the broader transformation toward intelligent and sustainable infrastructure materials. Realizing this vision will require not only algorithmic innovation but also a cultural and institutional shift toward open science, interdisciplinary collaboration, and digital sustainability. Through these collective efforts, AI-enabled coatings will play a defining role in shaping resilient, low-carbon, and adaptive infrastructures for the twenty-first century and beyond.

## 7. Conclusions

The evolution of polymeric coating science from empirically guided formulation to data-driven, AI-enabled engineering represents a significant shift in materials-design methodology. Traditional approaches—rooted in iterative experimentation and standardized testing—have provided the empirical foundation for understanding coating performance, yet remain limited by extended development timelines, substantial resource demands, and constrained predictive power.

The integration of artificial intelligence and machine learning is redefining this landscape by enabling predictive modeling, inverse design, and autonomous optimization as accelerated pathways for material discovery and performance evaluation. Through AI-assisted material selection, generative modeling, and hybrid data–physics frameworks, polymeric coatings can now be engineered with enhanced precision, establishing explicit linkages between molecular architecture and macroscopic durability, protective functionality, and sustainability.

When combined with DT platforms and SHM systems, intelligent coating technologies transcend their traditional role as passive protective layers to function as adaptive, self-aware elements within cyber-physical infrastructure. Such integration supports continuous monitoring, real-time degradation assessment, probabilistic forecasting, and condition-based maintenance strategies, offering a more proactive and efficiency-oriented alternative to conventional schedule-based inspection protocols.

The future development of AI-enabled coating systems will depend on robust data infrastructures, improved interpretability of machine-learning models, and rigorous validation through both laboratory and field testing. Emerging advances—such as autonomous experimentation environments, explainable AI methodologies, and environmentally informed design frameworks—are expected to further accelerate innovation while ensuring transparency, reliability, and ecological responsibility. Collectively, the convergence of AI, materials informatics, and digital engineering will enable a new generation of intelligent, self-optimizing, and sustainable polymeric coatings, reshaping both materials science and structural maintenance practices in support of resilient next-generation infrastructure.

## Figures and Tables

**Table 1:** Comparative Summary of Major Polymeric Coatings for Structural Applications [28,29,30,31,32,33].

Type	Chemical Structures/ Binder System	Primary Advantages	Typical Applications	Limitations	Refs.
Epoxy	Bisphenol A or Novolac epoxy + amine or anhydride hardener	Excellent adhesion, chemical resistance, hardness, and corrosion protection	Steel/concrete bridges, marine structures, and rebars in concrete	Brittle; poor UV resistance; chalking under sunlight	[28]
Polyurethane	Polyol + isocyanate crosslinking	High flexibility, abrasion resistance, UV stability, good gloss retention	Bridges, tanks, offshore platforms	Sensitive to humidity during curing	[29]
Acrylic	Poly (methyl methacrylate) or copolymers (e.g., styreneacrylate)	Transparency, weather resistance, color retention, environmental friendliness	Protective coatings for concrete facades and composite surfaces	Lower chemical and solvent resistance	[30]
Fluoropolymer	PVDF, PTFE, or FEVE-based polymers	Exceptional chemical resistance, low surface energy, self-cleaning	High-rise buildings, marine and chemical plants	Poor adhesion; high curing temperature; expensive	[31]
Silicone	Polysiloxane-based networks	Excellent thermal stability, hydrophobicity, anti-icing, and UV resistance	High-temperature surfaces, marine and anti-icing coatings	Limited mechanical strength; lower hardness	[32]
Hybrid/ Nanocomposite	Organic-inorganic networks (epoxy-silane, sol–gel, TiO_2_, SiO_2_, graphene fillers)	Synergistic strength, toughness, corrosion resistance, or self-healing behavior	Multifunctional marine coatings, self-healing and sensing layers	Complex synthesis; scale issues	[33]

**Table 2:** Some Representative Testing Methods in Traditional Coating Evaluation [34,35,36,37,38,39].

Property	Testing Method	Measurement	Refs.
Corrosion resistance	Salt spray (foggy) test	Time to rusting, blistering, or delamination	[34]
Moisture resistance	Humidity chamber test	Flim blistering, adhesion retention	[35]
Adhesion	Cross-cut, pull-off tests	Bond strength, interfacial failure pattern	[36]
Mechanical durability	Taber abrasion, impact resistance	Wear rate, deformation tolerance	[37]
Chemical resistance	Solvent hub, immersion tests	Surface degradation, color change	[38]
Weathering	QUV accelerated aging	Gloss retention, micro-cracking	[39]

**Table 3:** Relationship between industrial coating techniques and AI-predicted formulation parameters for structural applications [53,54,55,56,57,58,59].

Coating Technique	Typical Structural Use	Constraints	AI-Predicted Formulation Parameters	Mechanistic Influence on Application Success	Refs.
Airless spraying	- Steel bridges - Offshore structures - Wind turbine towers	- High shear during atomization - Risk of sagging on vertical surfaces - Nozzle clogging - Overspray and poor transfer efficiency	- Apparent viscosity - Solids content - Thixotropy index - Pot life/gel time	- Viscosity governs droplet breakup and spray stability - Excessively low viscosity - →Overspray and thin films - Excessively high viscosity - →Poor atomization and nozzle blockage - Thixotropy controls sag resistance after deposition	[53,54]
Roller coating	- Concrete floors, bridges, and tunnels - Confined spaces	- Limited shear energy - Manual application variability - Risk of roller marks and poor leveling	- Low-shear viscosity - Yield stress - Open time - Surface tension	- Yield stress must be low enough for roller transfer but high enough to prevent dripping - Open time controls re-rollability and surface uniformity - Surface tension affects wetting and defect formation	[55,56]
Dipping/ Immersion coating	- Small steel components - Prefabricated elements	- Drainage control - Film thickness governed by withdrawal speed - Risk of runs and pooling	- Newtonian viscosity - Solids content - Cure kinetics - Density	- Viscosity and solids content directly control wet-film thickness via gravitational drainage - Cure time must allow sufficient leveling before gelation - Rapid curing causes uneven films	[57,58]

**Table 4:** Summary of AI/ML algorithms in polymeric coatings.

AI/ML Algorithm	Typical Input	Applications in Polymeric Coatings	Key Strengths	Limitations
Convolutional Neural Networks	Images micrographs, hyperspectral data	Surface defect detection, coating thickness uniformity, microstructure analysis	Excellent spatial feature extraction; high accuracy in vision-based tasks	Requires large labeled datasets; low interpretability
Recurrent Neural Networks	Time-series data (aging, curing, degradation curves)	Cure kinetics modeling, durability and lifetime prediction	Captures temporal dependencies; suitable for degradation trends	Training instability; limited interpretability
Random Forests	Tabular data (formulation, processing, properties)	Property prediction, formulation optimization, screening studies	Robust to noise; good interpretability; works with small datasets	Limited extrapolation capability
Support Vector Machines	Low-to-medium dimensional structured data	Classification of coating performance, failure modes	Effective for small datasets; strong generalization	Limited scalability; kernel selection sensitivity
Artificial Neural Networks	Tabular, mixed experimental data	Property prediction, multi-parameter optimization	Flexible nonlinear mapping; widely used	Black-box nature; risk of overfitting
Graph Convolutional Networks	Molecular graphs, formulation networks	Polymer chemistry design, structure–property relationships	Encodes relational and molecular information effectively	High complexity; limited industrial datasets
Gaussian Process Regression	Small experimental datasets	Uncertainty-aware property prediction, Bayesian optimization	Provides uncertainty quantification; data-efficient	Poor scalability for large datasets

## Data Availability

No new data were created or analyzed in this study.

## Abbreviations

The following abbreviations are used in this manuscript:

AI	Artificial Intelligence
FRCs	Fiber-Reinforced Composites
UV	Ultraviolet
ML	Machine Learning
PVDF	Polyvinylidene Fluoride
FTIR	Fourier Transform Infrared
SEM	Scanning Electron Microscopy
DSC	Differential Scanning Calorimetry
TGA	Thermogravimetric Analysis
VOC	Volatile Organic Compound
GCNs	Graph Convolutional Networks
MPNNs	Message-Passing Neural Networks
VAEs	Variational Autoencoders
GANs	Generative Adversarial Networks
MD	Molecular Dynamics
FE	Finite Elements
LCA	Lifecycle Assessment
PIML	Physics-Informed Machine Learning
XAI	Explainable Artificial Intelligence
EIS	Electrochemical Impedance Spectroscopy
AE	Acoustic Emission
CNNs	Convolutional Neural Networks
RNNs	Recurrent Neural Networks
LSTM	Long Short-Term Memory
SHM	Structural Health Monitoring
RUL	Remaining Useful Life
IoT	Internet of Things
DT	Digital Twin
